# Homeless in the hospital: A call to strengthen multidisciplinary care for children awaiting out-of-home and psychiatric placements

**DOI:** 10.3389/fped.2022.1057956

**Published:** 2023-01-04

**Authors:** Aysha Jawed, Rachel Boro-Hernandez, Diane Pickett

**Affiliations:** ^1^ Department of Pediatric & OB/GYN Social Work, Johns Hopkins Children's Center, Baltimore, United States; ^2^ Department of Pediatric Social Work, Johns Hopkins Children's Center, Baltimore, United States

**Keywords:** pediatric, overstay, residential, out-of-home, psychiatry, placement

## Abstract

Increasingly more school-age and adolescent children continue to await psychiatric or out-of-home placements across pediatric healthcare systems. These children comprise the growing overstay pediatric population. The medical, developmental, socioemotional, behavioral and academic needs of these patients are complex and diverse. The uncertainty of waiting times for placement further continues. This growing pediatric issue emphasizes the importance of mobilizing a myriad of resources across healthcare and community contexts to support these patients during this precarious time. The reality is that there is a significant scarcity of placement resources which contributes to extended waiting times for placement. This contemporary issue brings child development, ethical and moral considerations, and healthcare operations to the forefront. We discuss a myriad of dimensions surrounding this growing issue from our clinical practice. We present a wealth of recommendations in working through each of these dimensions through a multi-systems approach that include development of individualized care plan, access to consistent psychiatric services, and implementing short-term and long-term goals pertaining to treatment and placement. We also review our clinical practices that have supported these patients on a continuum at our healthcare institution which integrate our recommendations and also involve an open line of communication with established community partners involved in the care of these children. Furthermore, we propose suggestions from an operational perspective on developing a comprehensive, multidisciplinary care model for this fragile and oftentimes neglected patient population across the healthcare system as the basis to achieve equity and translational impact in the quality and delivery of healthcare care services across pediatric healthcare systems.

## Introduction

The concept of stuck in the hospital when otherwise medically stable is one that has transcended across time – patients awaiting placement for rehabilitation, transition to another healthcare institution, inpatient psychiatric program, foster home amidst many more. However in contemporary times across pediatric healthcare systems, this concept has also taken on another layer of complexity for school-age and adolescent children with complex psychosocial circumstances.

Many of these children have complex and severe socioemotional, psychiatric, and behavioral diagnoses that require a higher level of care provided beyond the community outpatient context. Furthermore, a vast majority of the children have a significant history of trauma inclusive of child maltreatment. For children who necessitate admission to a residential treatment setting, their level of care and acuity extends beyond care that can safely be provided at home either with a biological caregiver, kinship care or foster family as well as in a group home setting (1). In many of these instances, the legal system has awarded the state jurisdiction with custody and guardianship of the child. In turn, the state is responsible in making placement decisions on behalf of the child.

Increasingly more hospitalized children are classified as overstays – patients on the waiting lists for out-of-home or psychiatric placement which could potentially last anywhere from weeks to nearly a year and possibly longer. A study conducted by the American Academic of Pediatrics revealed that nearly 40,000 to 66,000 children are boarding in hospitals amidst awaiting residential, group home or psychiatric placement on a yearly basis (2). It is important to note that group home settings and residential treatment facilities are also inclusive of psychiatric care. Not all psychiatric placements are long-term ones; however, group home and residential treatment placements are designed for lengthier inpatient treatment and further integrate medicine with psychiatry in the nature of provided treatment to children. The goal of this treatment is to address the severity of their socioemotional and psychiatric needs as the basis to achieve stabilization for this delicate population.

Of note, there is not ample descriptive literature available on the existing state-of-the-art which further suggests an incomplete understanding on how healthcare systems are addressing this pediatric issue. Based on recent data in the State of Maryland, approximately an average of 50 children meet the overstay criteria in awaiting out-of-home or psychiatric placement on a yearly basis (3). Of note at our healthcare institution, 81 children have met this criteria over the past year. It is important to note that in this specific US state, there are approximately 350 spaces across these facilities with increased waiting lists. The supply of these spaces significantly falls short of the demand. Ultimately increasingly more children are referred by social services to out-of-state facilities for evaluation and transition. It is also important to note that group home, residential treatment and psychiatric inpatient settings may have open spaces; however, the acuity in these spaces is substantially high which could also have a trickle down effect on time, energy, and resources invested by the placements and can in turn result in delayed acceptance of children to these placements.

The goals of this commentary are the following: (1) delineate a wealth of dimensions surrounding the care of overstay patients awaiting psychiatric and out-of-home placements in acute care settings, (2) describe strategies implemented in clinical practice to support these patients through a multi-systems approach, and (3) present a range of implications and recommendations for future research and practice with this growing pediatric population. Recommendations emerge from our vast clinical experiences as well as from the existing literature, especially with regard to the gap in research and practice across this trending pediatric issue.

## Level of care considerations

It is crucial to note that level of care is defined differently based on the psychiatric assessment of each patient. Oftentimes as a standard of care, the level of care is already established by the state before the child enters the hospital through recommendations from a prior psychiatric evaluation. In this instance, the existing level of care is accounted for in the child's inpatient care at the hospital. For example if there were significant concerns for suicide risk, self-harm in any other way, serious behavioral concerns, or elopement previously assessed by a state provider, these concerns will be integrated into the child's present treatment by implementing precautions for supervision through securing sitter coverage at all times.

However, sometimes a level of care is not already in existence for the child prior to hospitalization. In this specific instance, social work and psychiatry evaluates the child to ultimately determine whether there are any concerns for suicide risk, self-harm, aggressive/violent behaviors, and elopement as the basis to determine whether any of these precautions are necessary to implement for self-harm or flight risk.

There are also times when level of care is in place from state assessment but the care team at the hospital may have a different perspective on how the level of care could be implemented across the child's inpatient stay. For example, the multidisciplinary care team working with a child who is in shackles each time a healthcare provider enters the room may feel that these restrictions are dehumanizing the child in one or more ways. In another example, a child on elopement precautions may not be allowed to leave a room but a healthcare provider who has a rapport with the child could advocate that the child's quality of life will be optimized from reducing confinement in the same room space all the time during hospitalization. In both of these instances, recommendations from the care team are subject to change based on evolving assessments, rapport building, and weighing the child's needs, values, goals, and concerns amidst safety and liability considerations.

## Custody and guardianship circumstances

For most if not all of these children, a juridisdiction's Department of Social Services (DSS) has sole legal custody of these children. Under the concept of parens patriae, DSS bears the responsibility of protecting children in their custody by securing their safety and welfare at all times (4). Children are essentially wards of their designated state in the US. From a social services perspective if a child is in a safe place where their basic needs are met, this technically meets the expectation of social services in fulfilling their guardianship role. However given significant resource limitations that DSS often faces on a routine basis (e.g., turnover of staff, heightened caseloads), there could be variations in the degree of DSS involvement as the child's guardian in meeting basic needs of the child which could certainly impact length of hospital stay along with additional hospital-based factors while a child awaits placement in the hospital. In fact in these instances, the hospital ultimately emerges at the forefront in meeting all of the child's needs as DSS focuses their finite resources on identifying placement options. Furthermore, the hospital environment becomes the place of residence for these children. Although DSS is ultimately responsible for them, the reality is that the healthcare system interfaces with them significantly more during this time given that the children are in the physical care of the hospital. It follows that ultimately the hospital takes on responsibility of caring for these patients in these circumstances that extend beyond acute medical care.

## Mitigating inflammatory rhetoric

It is also crucial to account for the range of emotional responses from the care team surrounding prolonged hospitalization secondary to awaiting placement. Emotional responses can certainly be heightened during this time. Perspectives centered on stagnancy in a child's progress in global development oftentimes are prevalent and also raise ethical implications as well as concerns surrounding moral distress in caring for a child in the context of a subtherapeutic environment. In addition from an operational perspective, the notion that a hospital bed could have belonged to an acutely ill patient also is prevalent during prolonged hospitalizations attributed to placement delay. Each of these perspectives contributes towards generating inflammatory rhetoric around the uncertainty of transition which has implications to impair the child's multidisciplinary care throughout this vulnerable time.

In addition, it is imperative for the care team to maintain a united front with aligned goals to optimize meeting medical, socioemotional, psychological, developmental, academic and more needs during this prolonged time. Routine multidisciplinary team meetings (e.g., on a weekly basis) could heighten assurance and collaboration on establishing, reviewing, and redefining goals of care to meet the unique and complex needs of these patients consistently over the duration of their hospitalization. Further in a conscious effort to mitigate any inflammatory rhetoric that could arise, mobilizing support for the frontline staff could help address intense emotional responses in real time as the basis to promote positive coping and patient care to the highest standard for these patients.

## Individualized care plan

Based on our clinical experiences, one of our recommendations to begin optimizing care for children awaiting placement is to transition them out of the bustle of the emergency department onto a pediatric unit. The inpatient units promote a more healing environment given reduction in congestion prevalent in the emergency department. Our clinical impression is that this environmental change will yield more promise in developing and implementing care plans with the child and family.

Once a level of care for the child is established either from prior assessments or during hospitalization, we also recommend designing interventions with an individualized care plan for each child similar in concept to an individualized educational plan (IEP) that exists across school systems. Specifically developing a care plan that takes a biopsychosocial approach alongside respective stages of development will likely yield substantial promise in meeting the unique, diverse, and complex needs of these patients. Further in light of the fact that there is significant uncertainty in transition time to their next destination (a psychiatric or out-of-home placement), establishing this care plan with well-defined measurable goals over time could support each child in these circumstances to meet their global milestones respective of their stage of development, thereby reducing the risk of regression and other developmental losses. Furthermore, the care plan could also integrate a behavioral management plan that could assure ongoing safety assessments to ultimately determine the least restrictive measures to implement for the child over time as the basis to reward positive behaviors and optimize quality of life in the hospital. This care plan could also account for the child's socioemotional needs with respect to concerns surrounding abandonment, attachment/bonding, and social skills given the isolated nature of an unexpected hospitalization marked by uncertainty. Overall, a biopsychosocial approach in care planning can help account for the medical needs as well as a range of intrinsic and extrinsic factors centered on psychiatric, socioemotional, and environmental circumstances of each child on a continuum during waiting times for placement.

## Shared accountability across systems

One strategy that we have utilized in our practice is maintaining an open line of communication through weekly conference calls with outside agencies primarily DSS as well as the school system in coordinating care and understanding what is and is not working for each child on a routine basis. For children who are in between placements or children who are awaiting placement with no confirmed destination, ensuring that they are connected with their respective Home and Hospital teaching program through the designated jurisdiction will be the foundation for ensuring that their academic needs are met during their prolonged hospitalization. Working with DSS to help provide sitter services if they require 1:1 supervision, arranging for visitors to help them maintain a support network, and following up routinely on their plan of care towards securing placement are all measures that we recommend in optimizing this suboptimal waiting time for the child.

## Optimizing psychiatric care

Notably, mental disorders among children and adolescents placed in childhood welfare have been widely estimated at an average of 50% (5), representing nearly 4 times more than in the general population (6). All of these children have psychiatric and socioemotional needs which are not within the scope of acute treatment on a medical/surgical floor or in the pediatric emergency department. In addition to date, only a couple inpatient programs across pediatric healthcare systems account for the unique and diverse needs of these patients. In one healthcare institution that identified 437 cases of psychiatric boarding across pediatric medical units during a year, nearly 90% of children hospitalized on the medical units received brief supportive or cognitive behavioral therapy, 50% received psychotropic medication recommendations, and 27.5% had obtained behavioral plans from their care team (7). Another program specifically involves music therapy to support hospitalized children in strengthening coping skills in stabilization of behaviors while awaiting out-of-home or psychiatric placement (8).

Involving psychiatry and psychology teams from the onset of admission could help mobilize support to assess, refer, and provide psychiatric support for these children. From our clinical practice and in light of this growing pediatric issue in contemporary times, we recommend engaging institutional stakeholders in supporting the allocation of resources to equip pediatric healthcare systems in providing integrated medical and psychiatric care to our overstay pediatric population. Part of future work could account for expanding staffing and developing a care model for these inpatients across pediatric healthcare systems.

## Short-term and long-term care planning considerations

It is also important in these instances to take not only a short-term but also a long-term, global picture view of the complexity to establish realistic goals that can be achieved throughout the course of the child's prolonged and unknown duration of admission. The long-term plan could center on future placement in the background and the short-term plan could focus on the child's medical needs along with activities of daily living, global development, and academic needs. Another benefit of accounting for these considerations on a continuum throughout hospitalization could be to limit feelings of abandonment artificially created by awaiting placement. These feelings can be added to the mental suffering that motivated placement, thereby increasing artificially psychiatric symptoms within a vicious circle. It follows that addressing abandonment could be a predictive factor in increasing hope and making sense for patients to their circumstances.

Establishing goals of care certainly would entail getting all of the systems to work that are in place to serve the child (e.g., social services, healthcare system, and school system). Each of these systems shares accountability in supporting the child in one or more ways. Taking this approach for both short-term and long-term care planning yields potential in developing a streamlined coordinated approach as the basis to increase both assessment and intervention surrounding the complex needs of these patients.

## Conclusion

It is critical that the circumstances of these patients are visibly recognized and accompanied proactively through several adjustments in the existing care framework rather than remaining unthought and treated by default. However, adopting this adjusted position and subsequent practices could artificially extend this overstay crisis across pediatrics. For example, if psychiatric care providers and social services involved in the child's care are reassured about the benefits of pediatric hospitalization, it is possible that securing placement for the child may not be considered as high of a priority. It follows that this apprehension in healthcare may also explain limited applications so far of such relevant approaches in optimizing care for these patients that extend beyond lack of resources, competencies (for emergency healers to provide appropriate care to chronic situations in an acute unit previously not planned for such approach) and ethical concerns (limiting availability of acute beds).

Given the scarcity of existing literature on strategies, practices, and further interventions to optimize care and treatment for hospitalized children awaiting out-of-home or psychiatric placements, each of our recommendations seeks to optimize this critical window of time until the child transitions to their next destination. It follows that the constellation of each proposed recommendation could help provide a more complete therapeutic environment to meet the unique and diverse needs of these patients, thereby minimizing stagnancy in their global development and potentially contributing towards progress in their treatment.

## Data Availability

The original contributions presented in the study are included in the article/Supplementary Material, further inquiries can be directed to the corresponding author/s.
